# Time-Resolved Macromolecular Crystallography Frontiers at BioCARS: Serial Laue Micro-Crystallography and Electric Field Jump

**DOI:** 10.1063/4.0001016

**Published:** 2025-10-27

**Authors:** Robert Henning, Vukica Srajer, Irina Kosheleva, Insik Kim, Eric Zoellner, Rama Ranganathan

**Affiliations:** The University of Chicago, Chicago, IL, USA

## Abstract

BioCARS is a University of Chicago National User Facility with more than 25 years of scientific focus on synchrotron-based, dynamic studies in structural biology, initially using time-resolved protein crystallography and, more recently, time-resolved solution scattering. We are located at Sector 14, Advanced Photon Source, Argonne National Laboratory. Our 14-ID beamline uses two in-line undulators to provide high-flux pink- beam capability at 12 keV, with an adjustable bandpass between 2.5 - 5.5%. With a series of X-ray shutters, a single X-ray pulse can be isolated with up to ∼5.8 × 109 photons per pulse in 48-bunch mode and focused to 15 x 25 (V X H) μm2. We are equipped with ps and ns lasers (tunable from UV to IR) for reaction initiation in photosensitive samples or by temperature jump. With the ability to isolate single X-ray pulses, we can follow reactions in protein crystals in pump-probe experiments from 250ps to seconds. This poster focuses on more recent development of methods for time-resolved serial micro-crystallography at BioCARS, aimed at studies of irreversible reactions with minimized sample consumption and applicable to both photoinitiation and mix-and-inject approach. We also describe implementation of electric field jump as a novel method for reaction initiation in protein crystals.

